# Therapeutic potential of infrared-treated bee venom: enhanced multi-faceted bioactivity via compositional modulation

**DOI:** 10.1186/s40643-026-01026-3

**Published:** 2026-04-02

**Authors:** Husam Qanash, Aisha M. H. Al-Rajhi, Sulaiman A. Alsalamah, Abdulrahman S. Bazaid, Ali Alghubayshi, Safa H. Qahl, Amro Duhduh, Abadi M. Mashlawi, Mohamed M. Alawlaqi, Mashael Hakami

**Affiliations:** 1 Department of Medical Laboratory Science, College of Applied Medical Sciences, University of Ha’il, 55476 Hail, Saudi Arabia; 2 Medical and Diagnostic Research Center, University of Ha’il, 55473 Hail, Saudi Arabia; 3 Department of Biology, College of Science, Princess Nourah bint Abdulrahman University, P.O. Box 84428, 11671 Riyadh, Saudi Arabia; 4 Department of Biology, College of Science, Imam Mohammad Ibn Saud Islamic University (IMSIU), 11623 Riyadh, Saudi Arabia; 5 Department of Clinical Pharmacy, College of Pharmacy, University of Ha’il, 55473 Hail, Saudi Arabia; 6 Department of Biological Sciences, College of Science, University of Jeddah, P.O. Box 80327, 21959 Jeddah, Saudi Arabia; 7 Department of Medical Laboratory Technology, College of Nursing and Health Sciences, Jazan University, 45142 Jazan, Saudi Arabia; 8 Department of Biology, College of Science, Jazan University, P.O. Box 114, 45142 Jazan, Saudi Arabia; 9 Pharmacy Department, Jazan University Hospital, Jazan University, Jazan, Saudi Arabia

**Keywords:** Bee venom, Infrared irradiation, Antimicrobial, Antioxidant, Antibiofilm, Antihemolytic, Anti-inflammatory, Anticancer

## Abstract

Natural products are rich therapeutic sources, yet their translation into effective medicines remains challenging. Bee venom (BV) contains a diverse repertoire of bioactive molecules, but gentle, scalable methods to enhance its functionality are limited. This study, therefore, investigates a novel, solvent-free, post-extraction infrared (IR) conditioning step to fine-tune BV's composition and bioactivity. BV was irradiated (230 V, 50 Hz, 150 W) and compared to native extract using GC–MS and bioactivity assays. GC–MS revealed selective compositional tuning, with significant enrichment (*p* ≤ 0.05) of 4H-1-benzopyran-4-one, 2-(3,4-dimethoxyphenyl)-3,5-dihydroxy-7-methoxy. IR-treated BV exhibited enhanced antimicrobial activity, with increased zones of inhibition for *Staphylococcus aureus* (15 ± 0.1 vs. 11 ± 0.4 mm), *Bacillus subtilis* (24 ± 0.2 vs. 22 ± 0.6 mm), *Candida albicans* (26 ± 0.1 vs. 22 ± 0.5 mm), *Klebsiella pneumoniae* (24 ± 0.4 vs. 15 ± 0.3 mm), and *Salmonella typhi* (27 ± 0.8 vs. 20 ± 0.7 mm). MICs decreased for *S. aureus*, *B. subtilis*, and *K. pneumoniae*. The treated BV also showed stronger antibiofilm activity at 25% MBC concentrations for *K. pneumoniae* and *S. typhi* (*p* ≤ 0.05) and significantly reduced hemolysis at 25% MIC for *S. aureus* and *B. subtilis* (*p* ≤ 0.05). Antioxidant capacity increased (DPPH IC_50_: 16.47 ± 1.1 vs. 27.65 ± 0.8 µg/mL), as did anti-inflammatory activity (COX-2 IC_50_: 48.84 ± 0.2 vs. 50.99 ± 0.9 µg/mL; COX-1 IC_50_: 24.7 ± 0.2 vs. 41.74 ± 0.2 µg/mL). Cytotoxicity against PC-3 and SKOV-3 cells was maintained (IC_50_: 19.73 ± 0.9 and 19.76 ± 0.11 µg/mL, respectively, vs. native BV's 11.48 ± 0.3 and 17.46 ± 0.27 µg/mL). These findings establish brief IR irradiation as a practical, scalable post-processing strategy to selectively enhance the therapeutic potential of BV for biomedical applications.

**Graphical abstract:**

**Figure:**
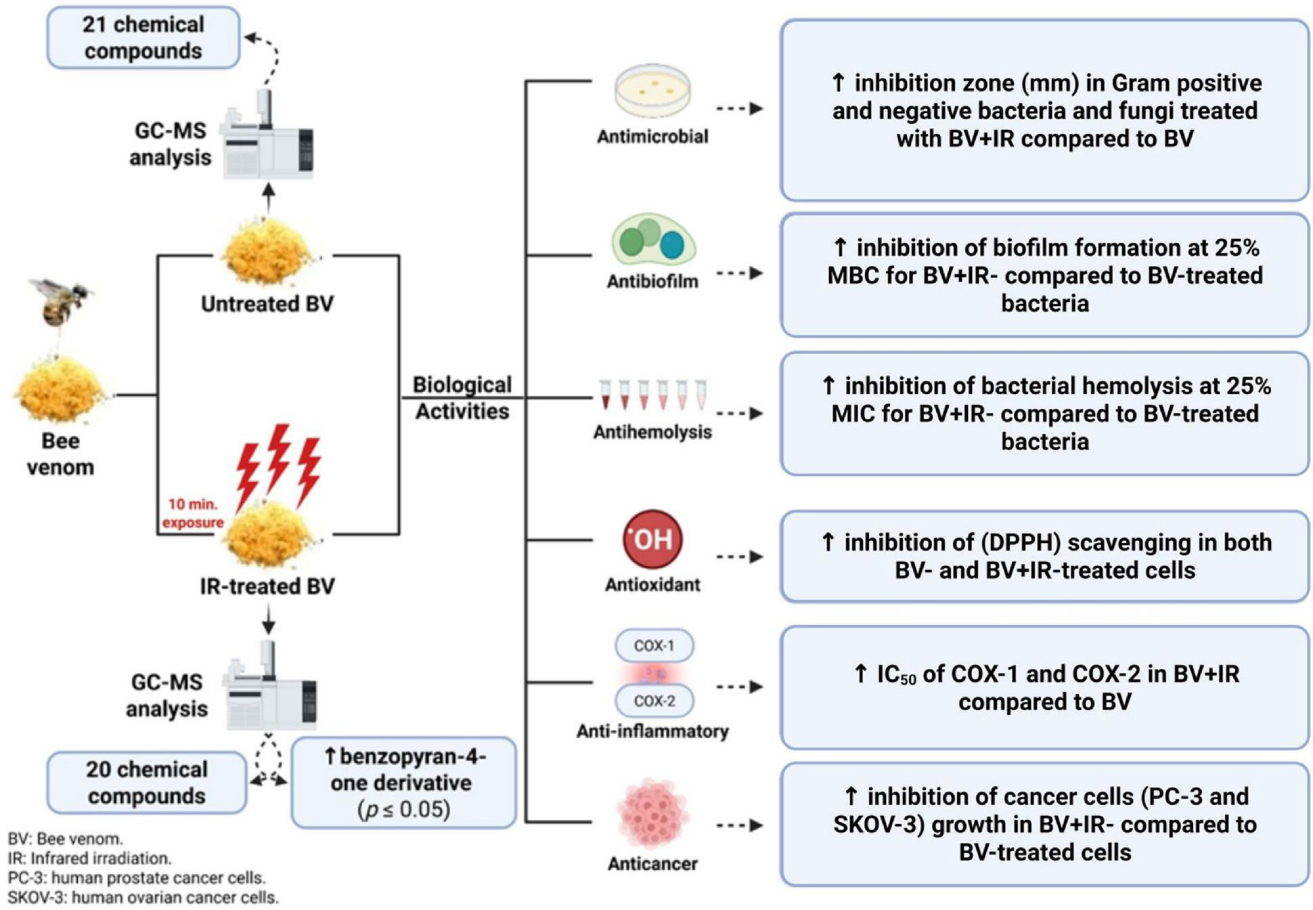
Created in BioRender. Qanash, H. (2026) https://BioRender.com/6war4d0

## Introduction

Natural compound-based therapies represent a significant source of agents that can complement conventional medical interventions for a wide spectrum of diseases. Honeybee venom (HBV), or apitoxin, is a complex biotoxin produced by the sting apparatus of honeybees (family Apidae) (Bava et al. 2023; Stela et al. 2024; Ullah et al. 2023). Beyond its primary role in predator defense, HBV is proposed to contribute to environmental antisepsis and social immunity within the colony (Seyam et al. 2022). Despite incomplete evidence regarding its efficacy and safety, bee-derived preparations are extensively utilized in traditional medicine for the prevention and treatment of various ailments (Elkomy et al. 2023; Pucca et al. 2019; Qanash et al. 2025a).

In the context of rising antimicrobial resistance, which increasingly compromises the efficacy of standard antibiotics, bee venom (BV) has emerged as a promising natural alternative with notable antimicrobial activity (El-Bilawy et al. 2025; Rabea et al. 2025). Its mechanism of action is attributed to a rich repertoire of peptides and enzymes, most notably melittin, phospholipase A_2_, apamin, and mast cell-degranulating peptides. These components can disrupt microbial membranes, depolarize cells, and interfere with intracellular targets, thereby reducing the likelihood of classical resistance development (Rady et al. 2017). While many microorganisms are beneficial, pathogenic species cause a broad spectrum of diseases in humans and animals, ranging from self-limiting conditions to life-threatening illnesses (El-Seedi et al. 2020; Fastl et al. 2023; Muteeb et al. 2023; Rahman et al. 2020). Furthermore, the capacity of bacterial pathogens to form biofilms significantly impairs antibiotic efficacy (Dodia et al. 2025). Notably, BV components have been reported to inhibit biofilm formation and disrupt established structures, suggesting their potential as adjuvants or surface-active antimicrobial agents (Shi et al. 2022).

Globally, the incidence and mortality of malignancies have risen over recent decades, imposing substantial health and economic burdens, particularly on aging populations (Zhu et al. 2024). This trend underscores the urgent need for novel preventive and therapeutic strategies, coupled with continued investment in anticancer research (Małek et al. 2023; Prathap et al. 2024; Yacoub et al. 2020). While modern medicine has improved disease management, curative outcomes remain elusive for many cancers. Consequently, there is growing interest in complementary approaches that utilize natural products from plants and animals for tumor suppression (Gajski et al. 2024; Rizvi et al. 2022). Constituents derived from BV have been investigated for their pro-apoptotic, anti-proliferative, and immunomodulatory effects relevant to oncology, alongside their well-documented anti-inflammatory actions (Ullah et al. 2023).

In many regions, conventional healthcare systems incorporate bee products, rich in natural antioxidants, as health adjuncts. Notably, BV exhibits immunomodulatory and anti-inflammatory properties, consistent with its role in the defensive mechanisms of bee colonies (Choudhary et al. 2023; El-Seedi et al. 2021; Kocyigit et al. 2019). The isolation and characterization of volatile fractions from BV typically employ techniques such as hydro-distillation for extraction and gas chromatography-mass spectrometry (GC–MS) for chemical profiling (Abd Elwahed et al. 2018; El Mehdi et al. 2022). However, the composition and bioactivity of BV are subject to variation influenced by botanical sources, seasonal factors, hive conditions, and post-collection handling (Carpena et al. 2020). Therefore, gentle and scalable post-processing methods that can standardize or enhance its functional profile without introducing solvents are highly desirable for translational applications.

This study investigates, for the first time, the effect of infrared (IR) irradiation on the chemical composition of BV using GC–MS. We also evaluate and compare the biological activities of IR-treated and untreated BV, including antimicrobial, antibiofilm, antioxidant, antihemolytic, anti-inflammatory, and anticancer properties. IR irradiation represents a brief, energy-efficient, and solvent-free intervention capable of modulating volatile and semi-volatile constituents. This approach offers a practical means of tailoring the properties of BV for diverse applications in human health.

## Materials and methods

### Collection and storage of bee venom (BV)

High-purity BV (apitoxin) was obtained from a beekeeper in Hail, Saudi Arabia, and stored at 4 °C until use. The collection method utilized an in-hive, lithium battery-powered device featuring closely spaced wires that deliver a mild electric stimulus to encourage venom deposition. To ensure purity, a glass plate covered with a food-grade shrink film was positioned beneath the wires to serve as the collection surface. The device was deployed in densely populated hives during periods of abundant foraging activity, characterized by high availability of pollen and nectar, to ensure the resulting venom met standard specifications (approximately 50% melittin by dry weight). The collector remained in the hive for 8–12 h before being removed and immediately transferred to a cool, shaded location. Throughout all handling procedures, beekeepers wore masks and sterile gloves to prevent contamination. The shrink film was subsequently peeled away, and the dried venom at the center of the plate was carefully scraped using a sterilized scalpel. Peripheral residues, which were typically contaminated with propolis and pollen grains, were discarded. The purified, dry venom was then transferred to sterile glass containers and stored frozen at −20 °C, a condition under which it remains stable for at least two years (Qanash et al. 2025a).

### Chemicals and infrared irradiation of BV

All chemical reagents, assay kits, and solvents were procured from Active Fine Chemicals Limited (Life Chemicals Group, El-Nozha, Cairo, Egypt). A 1.0 mg aliquot of dried BV was subjected to IR irradiation for 10 min using an infrared lamp (230 V, 50 Hz, 150 W; USA [Wavelength range: 1.4–3.0 µm, Emitter Temperature: (approx.) 500 °C]. Following irradiation, the BV sample was dissolved in 1.0 mL of dimethyl sulfoxide (DMSO) to prepare a stock solution with a concentration of 1,000 µg/mL.

### GC–MS/GC-FID analysis

GC–MS analysis was employed exclusively to profile volatile and semi-volatile low-molecular-weight constituents of bee venom. It is acknowledged that this technique does not allow for detection or characterization of peptide- or protein-based components, including major BV peptides such as melittin and apamin. The chemical compositions of non-irradiated and irradiated bee venom (BV) were analyzed by gas chromatography. Analyses were performed using a system equipped with a flame-ionization detector (FID) and an autosampler (Model 5810, PerkinElmer, Germany), coupled with an Rt-571 capillary column (100 m × 0.28 mm, 0.22 µm film thickness; Shimadzu, Japan). Data acquisition and processing were managed using X-caliber software (PerkinElmer, Germany). Helium served as the carrier gas at a constant flow rate of 1.7 mL/min, with a split injection mode at a 100:1 ratio. The pre-run and equilibration times were set to 10 min and 0.7 min, respectively. The oven temperature program was initiated at 49 °C, followed by a first ramp at 6 °C/min to 205 °C, a second ramp at 6 °C/min to 287 °C, and a final isothermal hold at 287 °C for 16.0 min. Volatile and semi-volatile components were identified by comparing their mass spectral data against the NIST 14 and 14 s mass spectral libraries (National Institute of Standards and Technology, USA). Quantitative analysis was performed using calibration curves that were prepared and corrected with internally generated standards (Alsalamah et al. 2025).

### Antimicrobial activity of non-irradiated and irradiated BV

The in vitro antibacterial and antifungal activities were assessed using the agar well diffusion method. Mueller–Hinton Agar (MHA) and Malt Extract Agar (MEA) were used as the growth media for bacterial and fungal strains, respectively. The plates were inoculated with 100 µL of standardized microbial suspensions (1.9 × 10^6^ CFU/mL for bacteria). Wells were created in the inoculated agar using a sterile cork borer and loaded with 100 µL of the respective BV sample. Gentamicin (0.08 mg/mL) and fluconazole (0.25 µg/mL) were used as standard antimicrobials, while 5.0% (v/v) DMSO served as the vehicle control. Incubation was carried out at 27 °C for 5 days for fungal assays and at 37 °C for 48 h for bacterial assays. Following incubation, the antimicrobial activity was quantified by measuring the diameter of the inhibition zones (including the well diameter) in millimeters (Al-Rajhi et al. 2025).

### Determination of minimum inhibitory concentration (MIC) and minimum bactericidal concentration (MBC)

The MIC was determined using the broth microdilution method in nutrient broth. Two-fold serial dilutions of the BV samples, ranging from 0.98 to 1,000 µg/mL, were prepared in 96-well microtiter plates (100 µL per well). Inocula were prepared from fresh microbial cultures and adjusted to a 1.0 McFarland standard; 2.5 µL of this suspension was added to each well using sterile 0.9% NaCl to achieve a final inoculum density of approximately 2.1 × 10^6^ CFU/mL. The plates were incubated at 35 ± 2 °C for 18–24 h for bacterial strains and at 27 °C for 5–7 days for fungal strains (Al-Rajhi et al. 2023a, b). The MIC was defined as the lowest concentration of the sample that completely inhibited visible microbial growth. Each experiment included a negative control (sterile medium) and appropriate positive reference controls (Al-Rajhi et al. 2023a, b). For the determination of the MBC, 100 µL aliquots from wells showing no visible growth were sub-cultured onto sterile nutrient agar plates. This included the growth control well and the test wells at and above the MIC. The MBC was defined as the lowest sample concentration that resulted in no growth on the subculture plates after incubation at the specified temperatures (Alawlaqi et al. 2023).

### Antibiofilm effects of non-irradiated and irradiated BV

The antibiofilm activity was evaluated using 96-well flat-bottom polystyrene plates. Each well received 280 µL of freshly prepared trypticase soy yeast broth, supplemented with 1% glucose, to achieve a final bacterial inoculum of 10^6^ CFU/mL. BV samples were tested at sub-inhibitory concentrations corresponding to 75%, 50%, and 25% of the predetermined MBC. Control wells included medium-only (sterility control) vehicle control (the solvent used for BV dissolution), and untreated inoculated wells (growth control). The plates were incubated statically at 37 °C for 48 h to facilitate biofilm formation. After incubation, the supernatant was carefully decanted, and the wells were gently washed twice with sterile distilled water to remove non-adherent cells. The plates were then air-dried for 30 min. The resulting biofilms were fixed and stained with 0.11% (w/v) crystal violet solution for 15 min at room temperature. Following staining, the wells were rinsed three times with sterile distilled water to remove excess dye. The bound crystal violet was subsequently solubilized by adding 250 µL of 97% ethanol to each well for 15 min. The absorbance of the solubilized dye was measured at 580 nm using a microplate reader to quantify biofilm biomass (Al-Rajhi et al. 2023b).

### Antihemolytic activity of non-irradiated and irradiated BV

3.0 mL sample of whole blood was drawn from a healthy human volunteer [after obtaining his informed consent and assure that the volunteer has a normal conation and don't has any hematologic diseases (Approval FSC22112024]. Blood transferred into heparinized vacuum tubes for erythrocyte isolation. Red blood cells (RBCs) were obtained by centrifugation at 3,000 × g for 10 min and washed three times with phosphate-buffered saline (PBS). The purified RBCs were resuspended to a 40% (v/v) concentration in 10.0 mM sodium phosphate buffer (pH 7.4). To assess the inhibition of bacterial hemolysin, the method of Rossignol et al. was followed (Rossignol et al. 2008). Bacterial cultures were standardized to an optical density (OD_600_) of 0.40 and then treated with sub-inhibitory concentrations of BV (25%, 50%, and 75% of the MIC); an untreated culture served as the hemolysin-producing control. Following incubation, the cultures were centrifuged at 23,000 × g for 17 min. A 500 µL aliquot of the cell-free supernatant was then mixed with 0.9 mL of a 2.0% (v/v) erythrocyte suspension in saline. The mixtures were incubated at 38 °C for 2 h and subsequently centrifuged at 13,000 × g for 12.0 min at 4 °C. Complete (100%) hemolysis was induced in a positive control using 0.10% sodium dodecyl sulfate, while erythrocytes incubated in LB broth served as the negative control. The percentage of hemolysis was calculated using the following formula:

$$ {\text{Hemolysis }}\left( \% \right) = \frac{{{\mathrm{OD}}\_{\mathrm{sample}} - {\mathrm{OD}}\_{\text{negative control}}}}{{{\mathrm{OD}}\_{\text{positive control}} - {\mathrm{OD}}\_{\text{negative control}}}} \times {\text{ }}100 $$

### Antioxidant activity of non-irradiated and irradiated BV

The free-radical scavenging activity was evaluated using the 1,1-diphenyl-2-picrylhydrazyl (DPPH) assay. Serial dilutions of the bee venom samples were prepared in ethanol to achieve a concentration range of 1.95–1000 µg/mL. A 3 mL aliquot of each concentration was mixed with 1 mL of a 0.1 mM DPPH solution in ethanol. The mixture was vortexed thoroughly and incubated in the dark at 27 °C for 35 min. The absorbance was subsequently measured at 525 nm using a UV–Vis spectrophotometer (Waters, USA). Ascorbic acid, prepared from a 40 µg/mL stock to yield a working range of 5–40 µg/mL, was used as a positive control. The percentage of DPPH scavenging activity was calculated using the following formula:

$$\begin{aligned} {\text{DPHH Scavenging Activity }}\left( \% \right) & \\ & = \frac{{{\mathrm{A}}\_{\mathrm{control}} - {\mathrm{A}}\_{\mathrm{sample}}}}{{{\mathrm{A}}\_{\mathrm{control}}}} \\ & \\ \end{aligned} \times100$$

where A_control is the absorbance of the DPPH solution with ethanol, and A_sample is the absorbance of the DPPH solution with the test sample. The IC_50_ value, defined as the concentration required to scavenge 50% of the DPPH radicals, was determined from the logarithmic regression of the dose–response curve (Abdel Ghany et al. 2019). IC₅₀ values were determined by logarithmic regression analysis of the dose–response curves (log concentration vs. percentage inhibition). Each assay was performed in triplicate (n = 3), and IC₅₀ values are reported as mean ± SD. The goodness of fit was evaluated using the coefficient of determination (R^2^), and 95% confidence intervals (CI) for IC₅₀ values were calculated using GraphPad Prism software.

### Anti-inflammatory activity (COX-1/COX-2 inhibition)

Cyclooxygenase (COX) inhibitory activity was assessed in vitro using commercial COX-1 and COX-2 inhibitor screening kits (Abcam, USA; catalog numbers ab204698 and ab267646, respectively), following the manufacturer's protocols. BV samples were prepared in 1.0% DMSO and tested across a concentration range of 0.5–1,000 µg/mL in a final assay volume of 1.0 mL. Celecoxib, reconstituted according to kit specifications, was used as the positive control. The IC_50_ values for both COX-1 and COX-2 inhibition were determined from non-linear regression analysis of the dose–response curves (Selim et al. 2025).

### Antitumor activity (MTT assay)

Cytotoxicity was evaluated against human prostate adenocarcinoma (PC-3) and human ovarian adenocarcinoma (SKOV-3) cell lines using the 3-(4,5-dimethylthiazol-2-yl)-2,5-diphenyltetrazolium bromide (MTT) colorimetric assay. An MTT stock solution was prepared by dissolving 5.0 mg of MTT in 1.0 mL of phosphate-buffered saline (PBS) to obtain a concentration of 5 mg/mL. Cells were seeded into 96-well plates at a density of 5 × 10^3^ cells/well in 100 µL of medium and incubated for 24 h at 37 °C under a 5% CO_2_ atmosphere to allow for adherence and monolayer formation. After the incubation period, the culture medium was aspirated, and the monolayers were washed twice with PBS. Serial dilutions of the BV samples, prepared in Human Plasma-like Medium supplemented with 2.0% serum, were then added to the wells (0.1 mL/well). Three wells on each plate containing only the vehicle served as the negative control. The plates were incubated at 37 °C and periodically inspected for morphological signs of toxicity. Following a 48-h exposure period, 20.0 µL of the MTT solution was added to each well, and the plates were gently agitated for 5 min at 160 rpm. They were then incubated for a further 4 h at 37 °C with 5% CO_2_ to allow for formazan crystal formation. The resulting formazan crystals were solubilized by adding 200 µL of DMSO to each well, followed by shaking for 5 min at 160 rpm. The optical density of the solution in each well was measured at 560 nm, with a reference wavelength of 620 nm used for background subtraction (Qanash et al. 2025b). The percentage of cell viability was calculated relative to the vehicle-treated control cells.

### Statistical analysis

All experiments were conducted with a minimum of three independent replicates (n ≥ 3), and data are expressed as the mean ± standard deviation. Statistical analyses were performed using GraphPad Prism software (Version 10, GraphPad Software Inc., San Diego, CA, USA). Comparisons between two groups were analyzed using an unpaired Student's t-test. For comparisons across multiple groups, a one-way analysis of variance (ANOVA) was employed. In all analyses, a *p*-value of less than 0.05 (*p* < 0.05) was considered statistically significant.

## Results and discussion

### Variations in volatile molecules after IR exposure

**Fig. 1:**
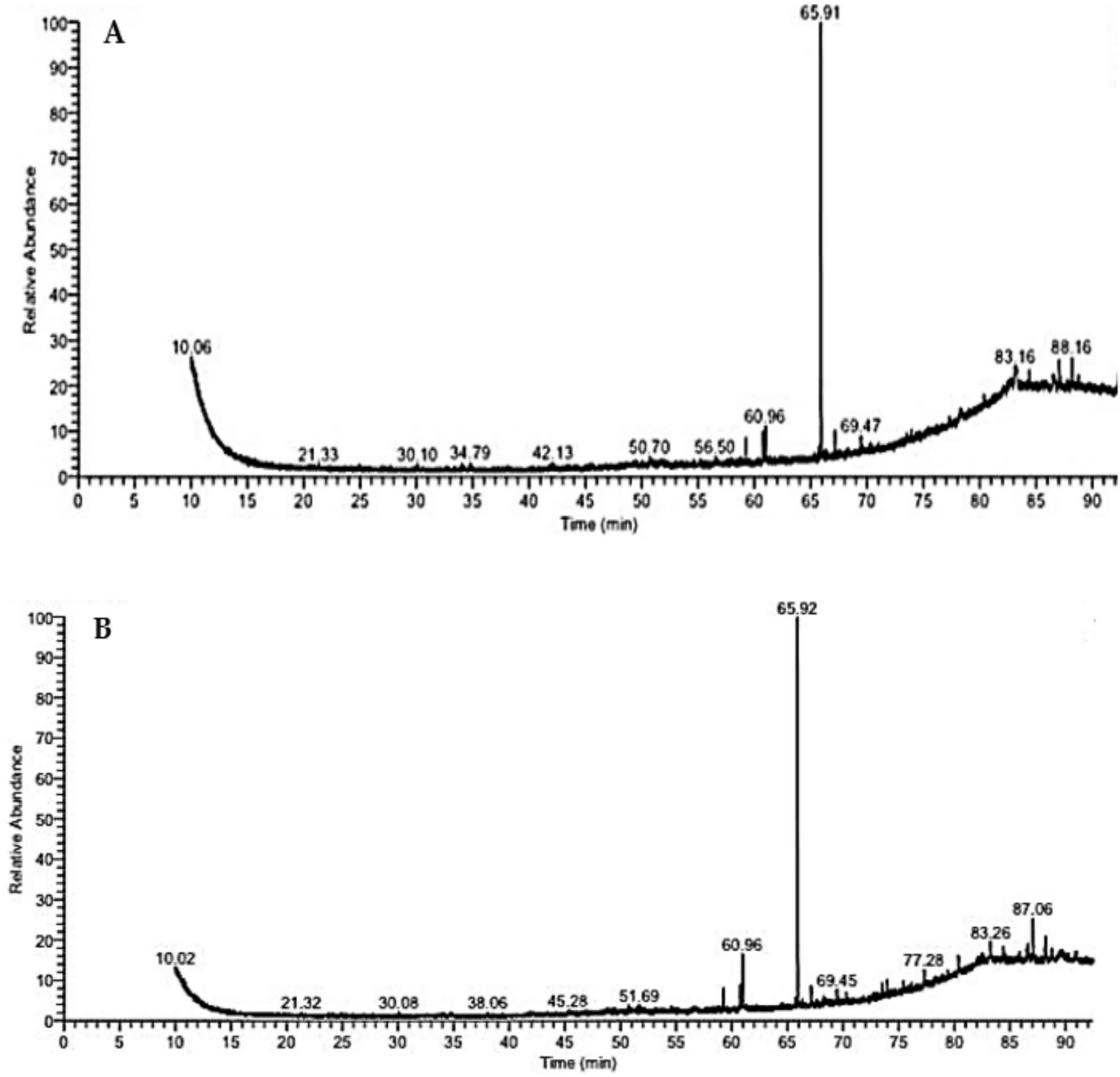
GC–MS profiles of volatile constituents in **A** bee venom (BV) extract and **B** infrared-irradiated BV extract

The compounds identified by GC–MS represent only the non-peptide, volatile/semi-volatile fraction of BV, and therefore do not reflect the full compositional profile of the venom, which is known to be dominated by bioactive peptides. In the present study, GC–MS analysis of the bee venom (BV) extract led to the identification of twenty-one chemical compounds, spanning twelve distinct classes: alkanes, fatty acid esters, phenols, fatty alcohols, steroids, fatty acids, organo-heterocyclic compounds, fatty aldehydes, fatty amides, sesquiterpene lactones, flavones, and phytosterols (Fig. 1A and Table 1). Following IR irradiation, the BV extract exhibited minimal qualitative alteration, with twenty compounds detected across ten classes. These included fatty acid esters, alkanes, steroids (including a steroidal carboxylic acid), sesquiterpene lactones, fatty acid amides, phenols, ketone oximes, flavones, acetylated sugar alcohols, and phytosterols (Fig. 1B and Table 1).

**Table 1:** Volatile constituents of bee venom (BV) identified by GC–MS, comparing the non-irradiated control with the infrared-irradiated sample (BV + IR)

BV						BV + IR					
Compound name	Molecular formula	Molecular weight	RT (min)	Area (%)	Class	Compound name	Molecular formula	Molecular weight	RT (min)	Area (%)	Class
n-Hexane	C_6_H_14_	86	10 0.06	1.14	Alkane	Eicosane	C_20_H_42_	282	30 0.08	0.42	Alkane
Eicosane	C_20_H_42_	282	30.10	1.05	Alkane	Estra-1,3,5(10)-trien-17 á-ol	C_18_H_24_O	256	50 0.70	0.67	Steroid
Tricosane	C_23_H_48_	324	34 0.02	1.05	Alkane	Octadecenoic acid, ethyl ester	C_20_H_40_O_2_	312	51 0.69	0.60	Fatty acid ester
2,4-Di-tert-butylphenol	C_14_H_22_O	206	34 0.79	0.77	Phenol	1-Eicosanol	C_20_H_42_O	298	54 0.57	0.61	Alkane
Pentacosane	C_25_H_52_	352	38 0.07	0.43	Alkane	Isochiapin-B	C_19_H_22_O_6_	346	56 0.51	0.63	Sesquiterpene lactone
14-β-H-Pregna	C_21_H_36_	288	49 0.29	0.5	Steroid	2-Hexadecanol	C_16_H_34_O	242	59 0.25	2.18	Fatty alcohol
Hexadecanoic acid	C_16_H_32_O_2_	256	50 0.70	1.24	Fatty acid	Myristamide, N-ethyl-	C_16_H_33_NO	255	60 0.74	2.74	Fatty acid amide
Octadecane, 3-ethyl-5-(2-ethylbutyl)-	C_26_H54	366	55 0.15	1.96	Alkane	11-Eicosenol	C_20_H_40_O	296	60 0.96	6.06	Alkane
Debrisoquine	C_10_H_13_N_3_	175	56 0.50	0.71	Organoheterocyclic compound	Phenol, 2,2’-methylenebis[6-(1 ,1-dimethyl ethyl)-4-m ethyl-	C_23_H_32_O_2_	340	65 0.92	48.07	Phenolic compound
4-Octadecenal	C_18_H_34_O	266	59 0.24	2.60	Fatty aldehyde	2-Nonadecanone, O-methyloxime	C_20_H_41_NO	311	67 0.12	2.14	Ketone oxime
Myristamide, N-ethyl-	C_16_H_33_NO	255	60 0.73	5.39	Fatty acid amide	Hexadecanoic acid, 2-(octadecyloxy)ethyl ester	C_36_H_72_O_3_	552	67 0.66	0.61	Fatty acid ester
Eicosen-1-ol, cis-9-	C_20_H_40_O	296	60 0.96	4.50	Fatty alcohol	Octadecane, 3-ethyl-5-(2-ethylbutyl)	C_26_H_54_	366	68 0.24	0.74	Alkane
2-Hexadecanol	C_16_H_34_O	242	65 0.69	2.66	Fatty alcohol	4 H-1-Benzopyran-4-one, 2-(3,4-dimethoxyphenyl)-3,5-dihydroxy-7-methoxy	C_18_H_16_O_7_	344	69.45	14.05	Flavone
Phenol, 2,2’-methylenebis[6-(1 ,1-dimethyl ethyl)-4-m ethyl-	C_23_H_32_O_2_	340	65 0.91	47.08	Phenolic compound	Propanoic acid, 2-(3-acetoxy-4,4,14-trim ethylandrost-8-en-17-yl)	C_27_H_42_O_4_	430	73 0.95	2.93	Steroidal carboxylic acid
Isochiapin-B	C_19_H_22_O_6_	346	69 0.47	1.71	Sesquiterpene lactone	Oleic acid, 3-(octadecyloxy)propyl ester	C_39_H_76_O_3_	592	77 0.28	2.48	Fatty acid ester
4 H-1-Benzopyran-4-one, 2-(3,4-dimethoxyphenyl)-3,5-dihydroxy-7-methoxy	C_18_H_16_O_7_	344	73 0.94	7.74	Flavone	D- Xylitol pentacetate	C_15_H_22_O_10_	362	82 0.11	0.68	Acetylated sugar alcohol
Oleic acid, 3-(octadecyloxy)propyl ester	C_39_H_76_O_3_	592	80 0.35	2.14	Fatty acid ester	stearic acid, 3-(octadecyloxy)propyl ester	C_39_H_78_O_3_	594	83 0.26	0.68	Fatty acid ester
Spirost-8-en-11-one, 3-hydroxy-, (3á,5à,14á,20á,22á,25)	C_27_H_40_O_4_	428	83 0.16	6.90	Steroid	Spirost-8-en-11-one, 3-hydroxy-, (3á,5à,14á,20á,22á,25)	C_27_H_40_O_4_	428	84 0.39	3.60	Steroid
stearic acid, 3-(octadecyloxy)propyl ester	C_39_H_78_O_3_	594	86 0.46	2.99	Fatty acid ester	Oleic acid, eicosyl ester	C_38_H_74_O_2_	562	87 0.06	6.64	Fatty acid ester
Oleic acid, eicosyl ester	C_38_H_74_O_2_	562	87 0.06	3.55	Fatty acid ester	β-Sitosterol	C_29_H_50_O	414	88 0.19	3.47	Phytosterol
β -Sitosterol	C_29_H_50_O	414	88 0.16	3.89	Phytosterol						

Furthermore, twelve compounds were common to both the untreated BV and IR-treated BV extracts. These shared compounds were eicosane; octadecane, 3-ethyl-5-(2-ethylbutyl)-; N-ethylmyristamide; 2-hexadecanol; phenol, 2,2′-methylenebis[6-(1,1-dimethyl ethyl)-4-methyl]-; isochiapin B; 4H-1-benzopyran-4-one, 2-(3,4-dimethoxyphenyl)-3,5-dihydroxy-7-methoxy; oleic acid, 3-(octadecyloxy)propyl ester; spirost-8-en-11-one, 3-hydroxy-, (3α,5α,14α,20α,22α,25R); stearic acid, 3-(octadecyloxy)propyl ester; oleic acid, eicosyl ester; and β-sitosterol (Table 1). Two major constituents, including phenol, 2,2′-methylenebis[6-(1,1-dimethyl ethyl)-4-methyl]- and 4H-1-benzopyran-4-one, 2-(3,4-dimethoxyphenyl)-3,5-dihydroxy-7-methoxy, were present in both extracts but at different concentrations. Specifically, the phenolic dimer exhibited a slight increase after IR irradiation, whereas the benzopyran-4-one derivative increased significantly (*p* ≤ 0.05). Conversely, five compounds, such as eicosane; octadecane, 3-ethyl-5-(2-ethylbutyl)-; N-ethylmyristamide; spirost-8-en-11-one, 3-hydroxy-, (3α,5α,14α,20α,22α,25R); and stearic acid, 3-(octadecyloxy)propyl ester, were significantly decreased following IR treatment (*p* ≤ 0.05) (Table 1). In contrast, four compounds, including 2-hexadecanol; oleic acid, 3-(octadecyloxy)propyl ester; oleic acid, eicosyl ester; and β-sitosterol, showed no significant change between the two extracts (Table 1).

BV comprises a diverse array of volatile molecules, including alcohols, acid esters, terpenoids, esters, aldehydes, ketones, and hydrocarbons, synthesized by the venom glands. These compounds contribute to its overall composition and bioactivity, often serving as alarm pheromones (Wehbe et al. 2019). It is well-established that volatile profiles can vary considerably among different bee populations (Isidorov et al. 2023; Montaser et al. 2023). Infrared radiation, defined as electromagnetic energy with wavelengths just below visible red light, is known to induce molecular vibrations, generate internal heating, enhance drying processes, and improve energy efficiency. These effects can facilitate extraction and potentially modify volatile profiles (Abbaspour-Gilandeh et al. 2021; Sabbaghi & Nguyen 2025; Togrul 2005). Taken together, our data indicates that brief IR exposure modulates the BV volatilome, significantly enriching a specific benzopyran-4-one derivative without substantially altering the overall number of detected compounds.

### Antimicrobial efficacy and MIC levels

**Fig. 2:**
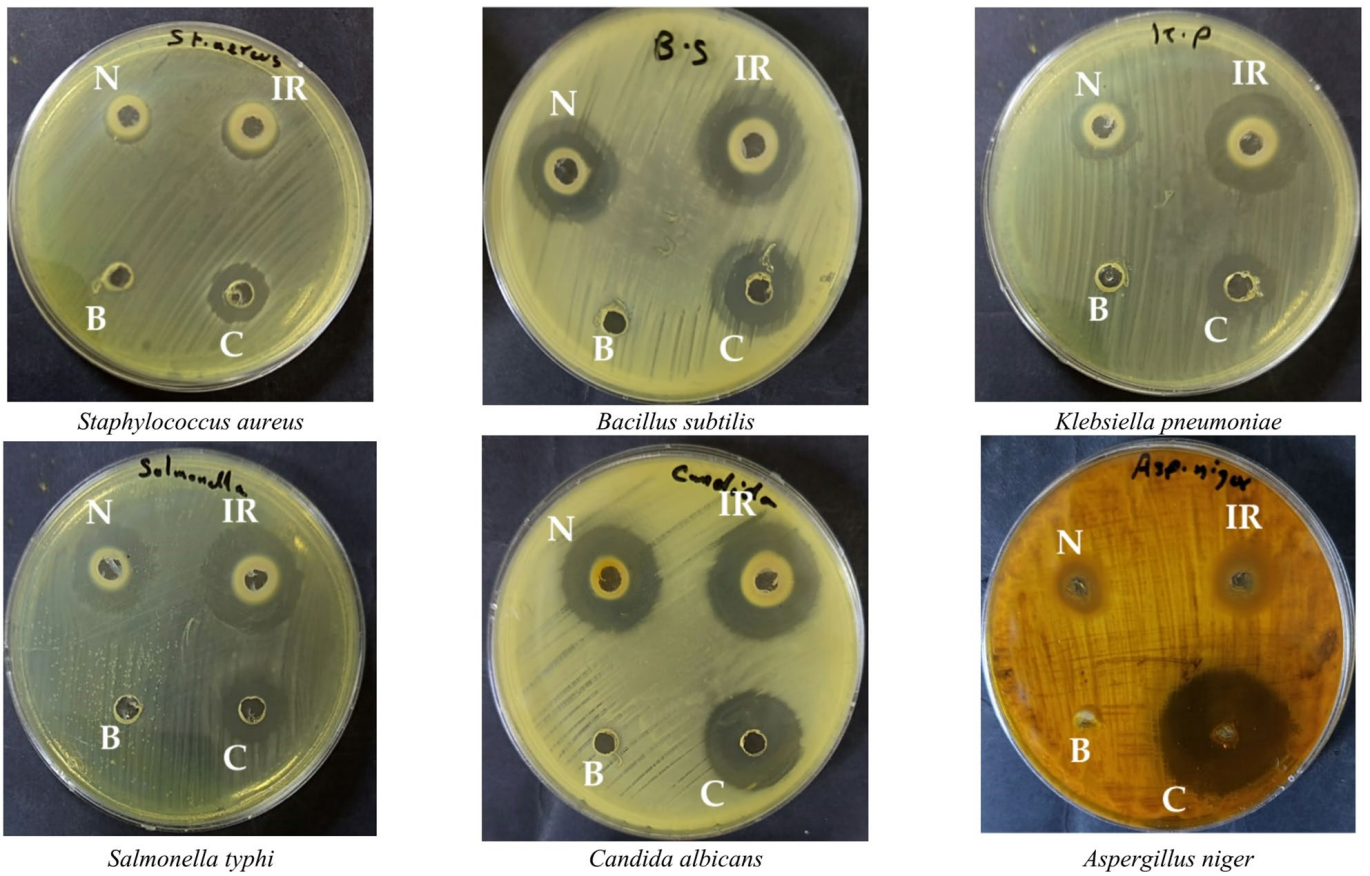
Agar well diffusion assay showing antimicrobial activity of tested samples against representative Gram-positive, Gram-negative bacteria, and molds. Labels: **N** = bee venom (non-irradiated), **IR** = infrared-irradiated bee venom, **B** = blank (vehicle control), **C** = standard drug. Inhibition zones (mm) indicate activity [Plate diameter: 150 mm petri dishes, well diameter: approximately 6 mm, the zone of inhibition is measured as the entire diameter of the clear area, including the central well, inoculum density per plate: 0.5 McFarland standard]

BV demonstrated promising antimicrobial activity compared to standard drugs, though it exhibited no inhibitory effect against *Aspergillus niger*. IR irradiation moderately enhanced this activity against *Staphylococcus aureus*, *Bacillus subtilis*, and *Candida albicans*, with inhibition zones increasing from 11 ± 0.4, 22 ± 0.6, and 22 ± 0.5 mm to 15 ± 0.1, 24 ± 0.2, and 26 ± 0.1 mm, respectively (Fig. 2 and Table 2). A more pronounced enhancement was observed against *Klebsiella pneumoniae* and *Salmonella typhi*, where the inhibition zones increased from 15 ± 0.3 and 20 ± 0.7 mm to 24 ± 0.4 and 27 ± 0.8 mm, respectively.

**Table 2:** Antimicrobial examination (mm) of BV control and after exposure to IR using agar diffusion test. Data are tabulated as means ± SD

Microorganism	Sample		
	BV (mm)	BV + IR (mm)	Standard drug (mm)
*S. aureus* (ATCC 6538)	11 ± 0.4	15 ± 0.1	14 ± 0.3
*B. subtilis* (ATCC 6633)	22 ± 0.6	24 ± 0.2	20 ± 0.6
*K. pneumoniae (*ATCC13883)	15 ± 0.3	24 ± 0.4	15 ± 0.5
*S. typhi* (ATCC 6539)	20 ± 0.7	27 ± 0.8	15 ± 0.3
*C. albicans* (ATCC 10221)	22 ± 0.5	26 ± 0.1	23 ± 0.8
*A. niger* (ATCC16888)	NA	NA	31 ± 1.0

Consistent with these findings, the MIC values decreased following IR exposure. The MIC for *S. aureus*, *B. subtilis*, and *K. pneumoniae* decreased from 31.25 ± 0.2, 62.50 ± 0.5, and 62.50 ± 0.4 µg/mL to 15.62 ± 0.5, 31.25 ± 0.4, and 7.80 ± 0.6 µg/mL, respectively. Further reductions were recorded for *S. typhi* and *C. albicans*, with MICs declining from 31.25 ± 0.3 and 31.25 ± 0.6 µg/mL to 7.80 ± 0.3 and 15.62 ± 0.2 µg/mL, respectively (Table 3).

**Table 3:** Minimum inhibitory concentrations (MIC; µg/mL) of bee venom (BV) before and after infrared (IR) irradiation. Data are reported as mean ± SD

Microorganism	Sample#	
	BV (µg/mL)	BV + IR (µg/mL)
*S. aureus* (ATCC 6538)	31.25 ± 0.2	15.62 ± 0.5*
*B. subtilis* (ATCC 6633)	62.50 ± 0.5	31.25 ± 0.4*
*K. pneumoniae (*ATCC13883)	62.50 ± 0.4	7.80 ± 0.6***
*S. typhi* (ATCC 6539)	31.25 ± 0.3	7.80 ± 0.3**
*C. albicans* (ATCC 10221)	31.25 ± 0.6	15.62 ± 0.2*

^***#***^ [analysis done by One-way Analysis of Variance (ANOVA), followed by post-hoc test where *P ≤ 0.05, **P ≤ 0.01, ***P ≤ 0.001]

Interest in BV as a source of natural antimicrobial agents has intensified in the context of rising antibiotic resistance, given its documented capacity to inhibit both Gram-positive and Gram-negative bacteria as well as certain fungi (Maitip et al. 2021; Pérez-Delgado et al. 2023). The observed enhancement in efficacy post-IR irradiation may be attributed to several mechanisms, including an increased yield of bioactive compounds, improved penetration of microbial cells, or direct damage to microbial structures. The specific outcomes of such treatments are known to depend on factors including the extract type, target microorganism, and the parameters of the IR radiation, such as wavelength, intensity, and exposure duration (Gonzalez-Pastor et al. 2023; Hammoud et al. 2022; Nortjie et al. 2024). Collectively, the results of this study suggest a link between the IR-induced compositional shifts detailed earlier and a selective enhancement of antimicrobial performance, as evidenced by the enlarged zones of inhibition and reduced MIC values.

### Effect of IR irradiation on antibiofilm activity

**Fig. 3:**
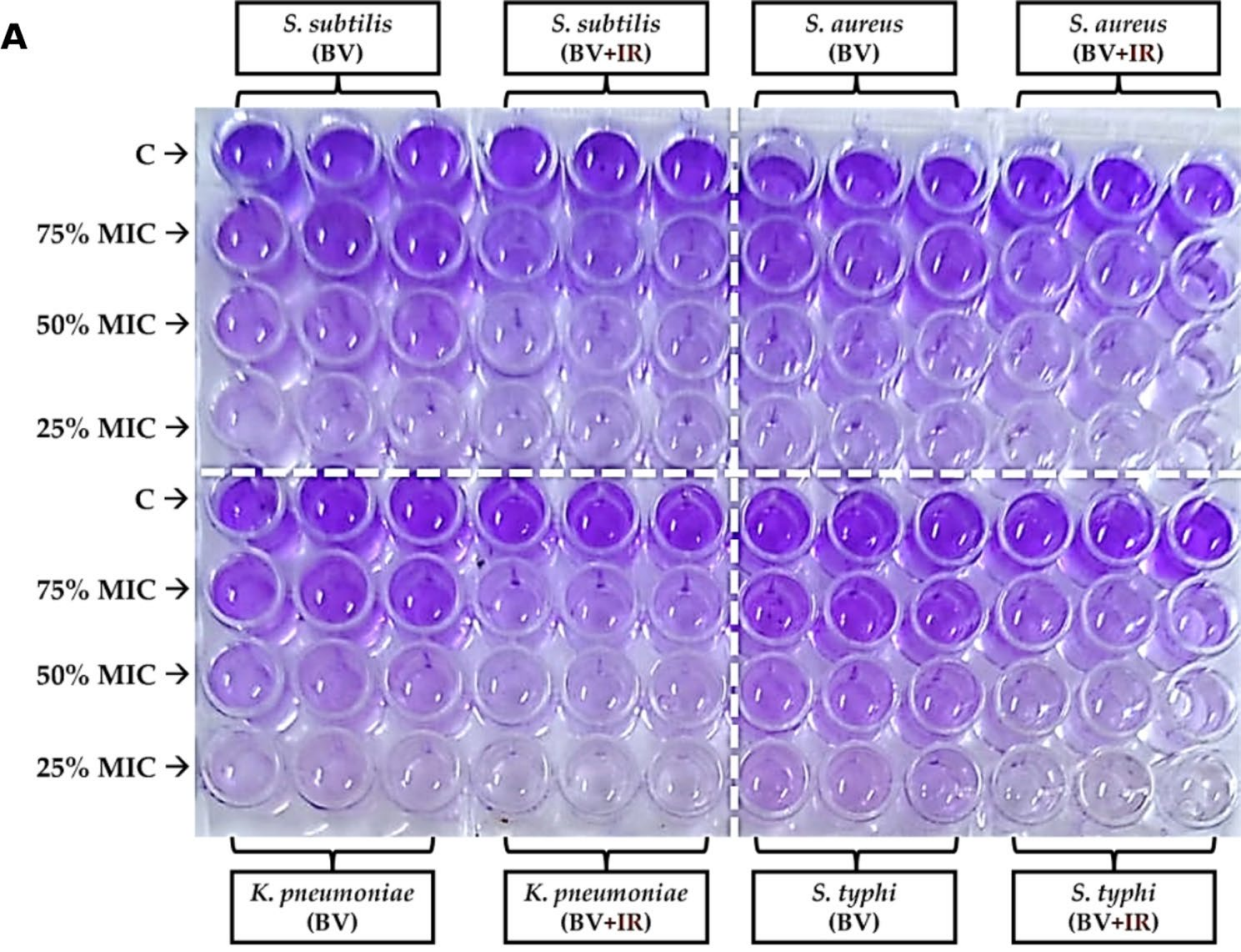

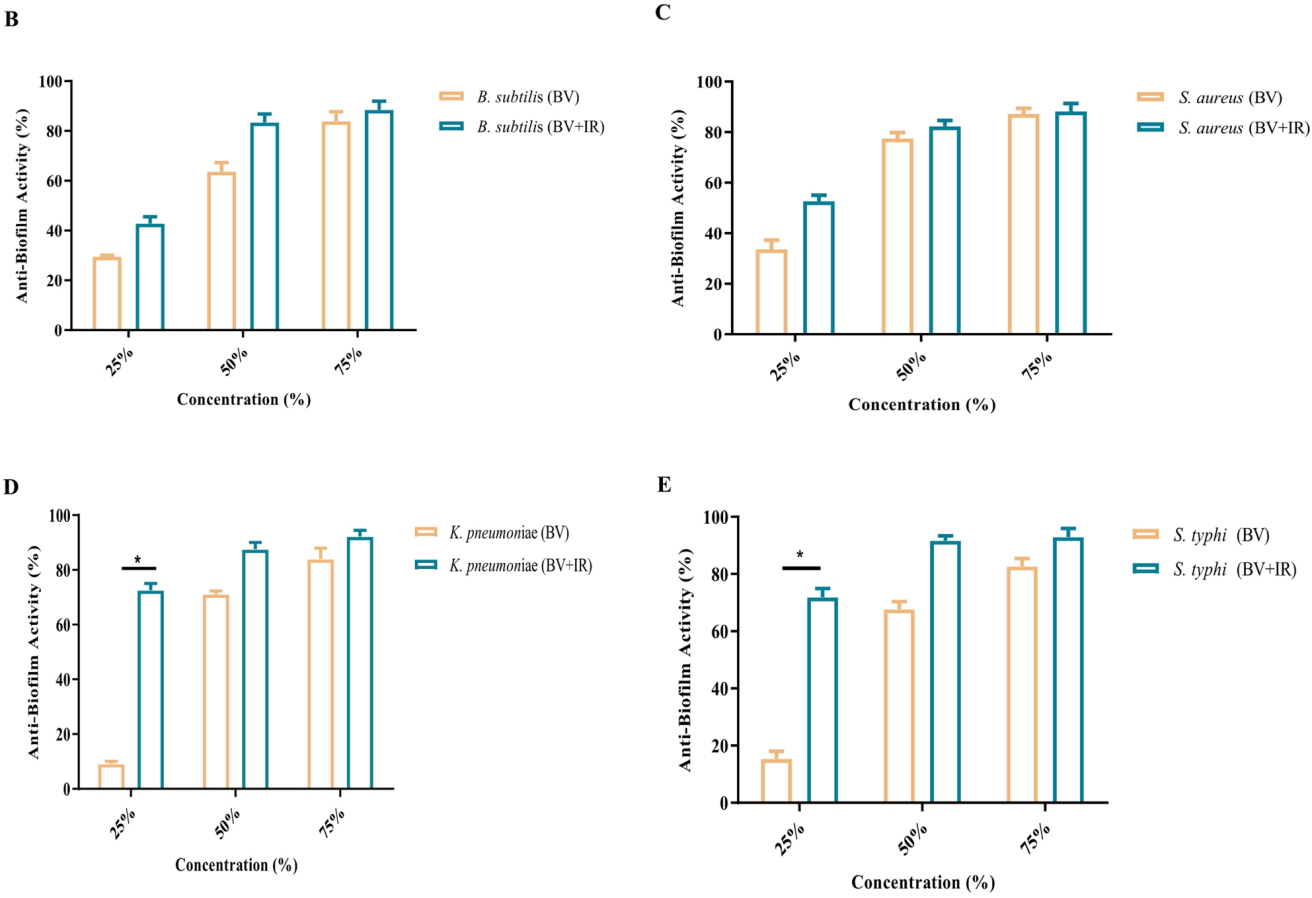
Antibiofilm activity of bee venom (BV) and infrared-irradiated BV against representative bacteria. **A** Microplate layout showing treatment conditions. **B**–**E** Quantitative antibiofilm analyses for **B** *Bacillus subtilis*, **C** *Staphylococcus aureus*, **D** *Klebsiella pneumoniae*, and **E** *Salmonella typhi*. Data are reported as mean ± SD (n = 3); [analysis done by One-way Analysis of Variance (ANOVA), followed by post-hoc test * indicates *p* ≤ 0.05

The antibiofilm activity of BV was significantly enhanced following IR irradiation. A maximal inhibition of biofilm formation was observed against *B. subtilis* and *S. aureus* at a concentration of 75% of the MBC for the IR-treated BV compared to the control. A significant increase (*p* ≤ 0.05) in activity was also recorded at 25% of the MBC for the irradiated BV against *K. pneumoniae* and *S. typhi* when compared to the non-irradiated BV. For these pathogens, further improvements in biofilm inhibition were achieved at concentrations of 50% and 75% of the MBC (Fig. 3).

BV is recognized for its capacity to disrupt pre-formed biofilms and inhibit their formation, typically demonstrating greater efficacy in the disruption of mature biofilms than in preventing their initial development (Awad et al. 2025). Infrared-assisted extraction can potentiate these antibiofilm effects, likely by increasing the concentration of key bioactive constituents; it may also exert a direct influence on biofilm matrix integrity (Lin et al. 2021; Luzala et al. 2022). Notably, the findings from this study position IR-processed BV as an effective biofilm-disrupting agent, capable of operating at sublethal concentrations across both Gram-positive and Gram-negative bacterial models.

### Effect of IR irradiation on hemolysis inhibition

**Fig. 4:**
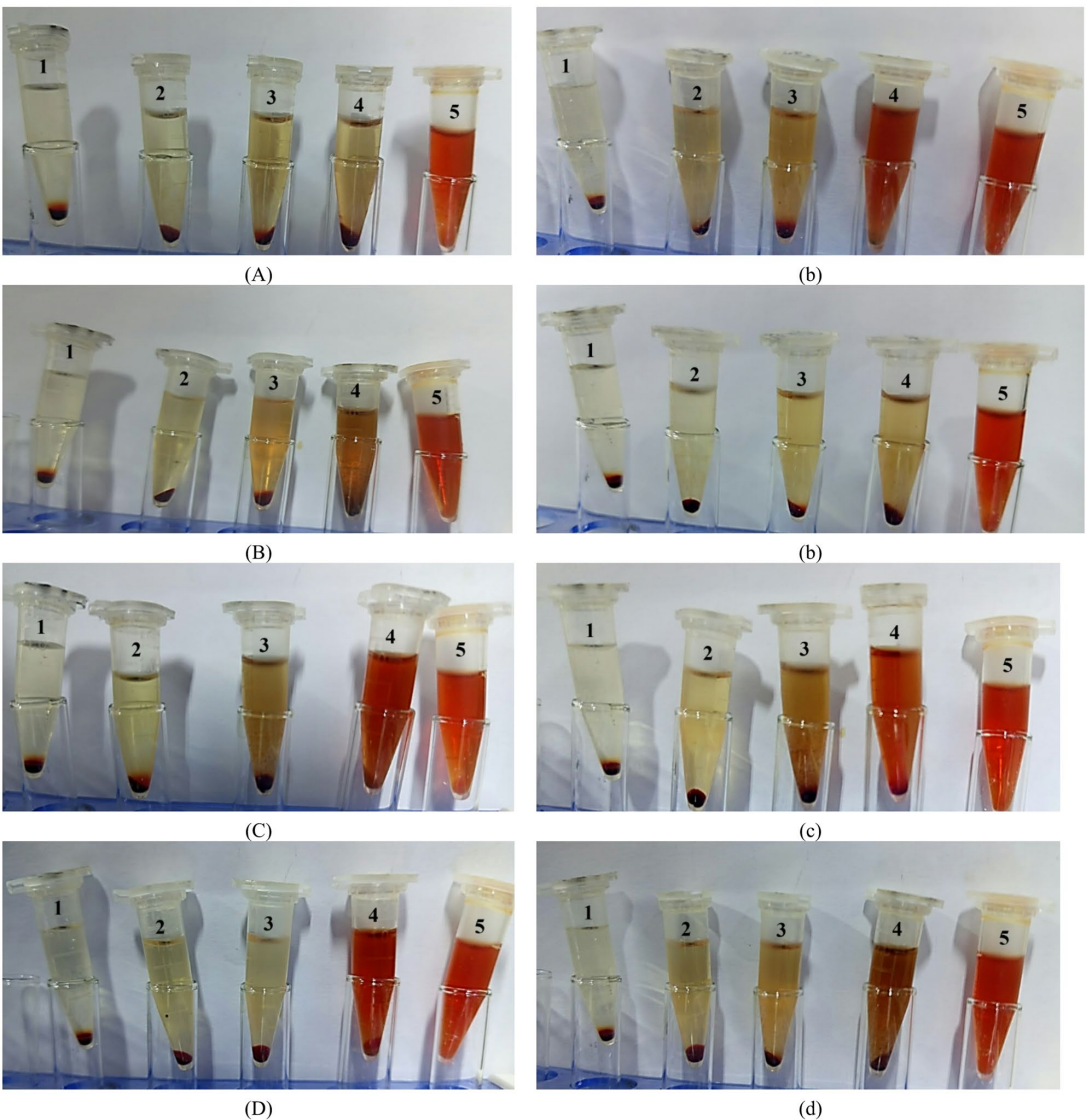
The hemolysis inhibition in the presence of **A**;**a** *Staphylococcus aureus*, **B**;**b** *Bacillus subtilis*, **C**;**c** *Klebsiella pneumoniae*, and **D**;**d** *Salmonella typhi* with non-irradiated bee venom (BV) (left panel, uppercase labels) and infrared-irradiated BV (BV + IR) (right panel, lowercase labels). Treatments: **1** standard drug, **2** 25% MIC, **3** 50% MIC, **4** 75% MIC, and **5** untreated

**Fig. 5:**
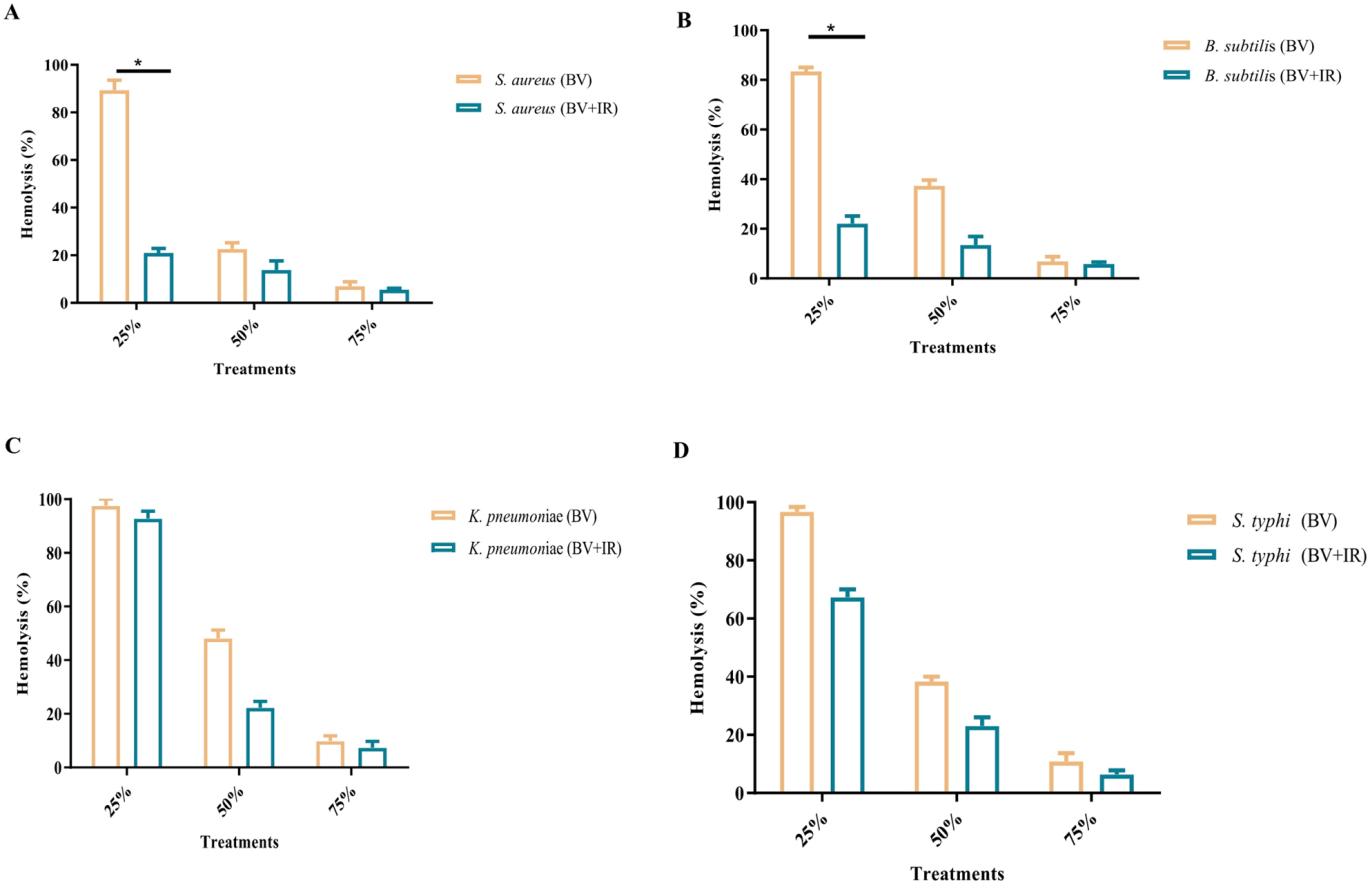
Antihemolytic activity (%) of bee venom (BV) formulations against **A** *Staphylococcus aureus*, **B** *Bacillus subtilis*, **C** *Klebsiella pneumoniae*, and **D** *Salmonella typhi*. Data are reported as mean ± SD; [analysis done by One-way Analysis of Variance (ANOVA), followed by post-hoc test * denotes significant difference at *p* ≤ 0.05

The inhibition of hemolysis was evaluated for both native and IR-irradiated BV in the presence of bacteria at sub-MIC levels (Figs. 4 and 5). At a concentration corresponding to 25% of the MIC, the irradiated BV demonstrated a significantly lower capacity to inhibit bacterial hemolysin-mediated red blood cell lysis, resulting in increased hemolysis compared to native BV in the presence of S. aureus and B. subtilis (*p* ≤ 0.05). More moderate decreases in hemolysis inhibition (i.e., partial loss of antihemolytic protection) were also observed at 50% and 75% of the MIC for these species.

In contrast, for *K. pneumoniae* and *S. typhi*, IR-irradiated BV caused only marginal increases in hemolysis, indicating largely preserved antihemolytic activity relative to the non-irradiated extract across the tested sub-MIC levels (25%, 50%, and 75%).

Although BV itself can be hemolytic, certain volatile constituents have been reported to mitigate red blood cell lysis (Bava et al. 2023). The effect of IR exposure on this property is variable; it can either attenuate or exacerbate hemolytic capacity depending on the compositional changes induced and the specific experimental conditions. In some contexts, IR treatment may reduce oxidative stress and protect erythrocytes, whereas in others it can promote hemolysis (Aboud et al. 2019; Alecu et al. 2025; Kumar et al. 2024). These divergent effects are likely attributable to IR-induced alterations in the profile and bioactivity of specific compounds. Collectively, our findings indicate that IR irradiation serves as a tool for adjusting the safety-efficacy profile of BV, producing pathogen- and dose-dependent modulations in hemolysis inhibition.

### Antioxidant activity

**Fig. 6:**
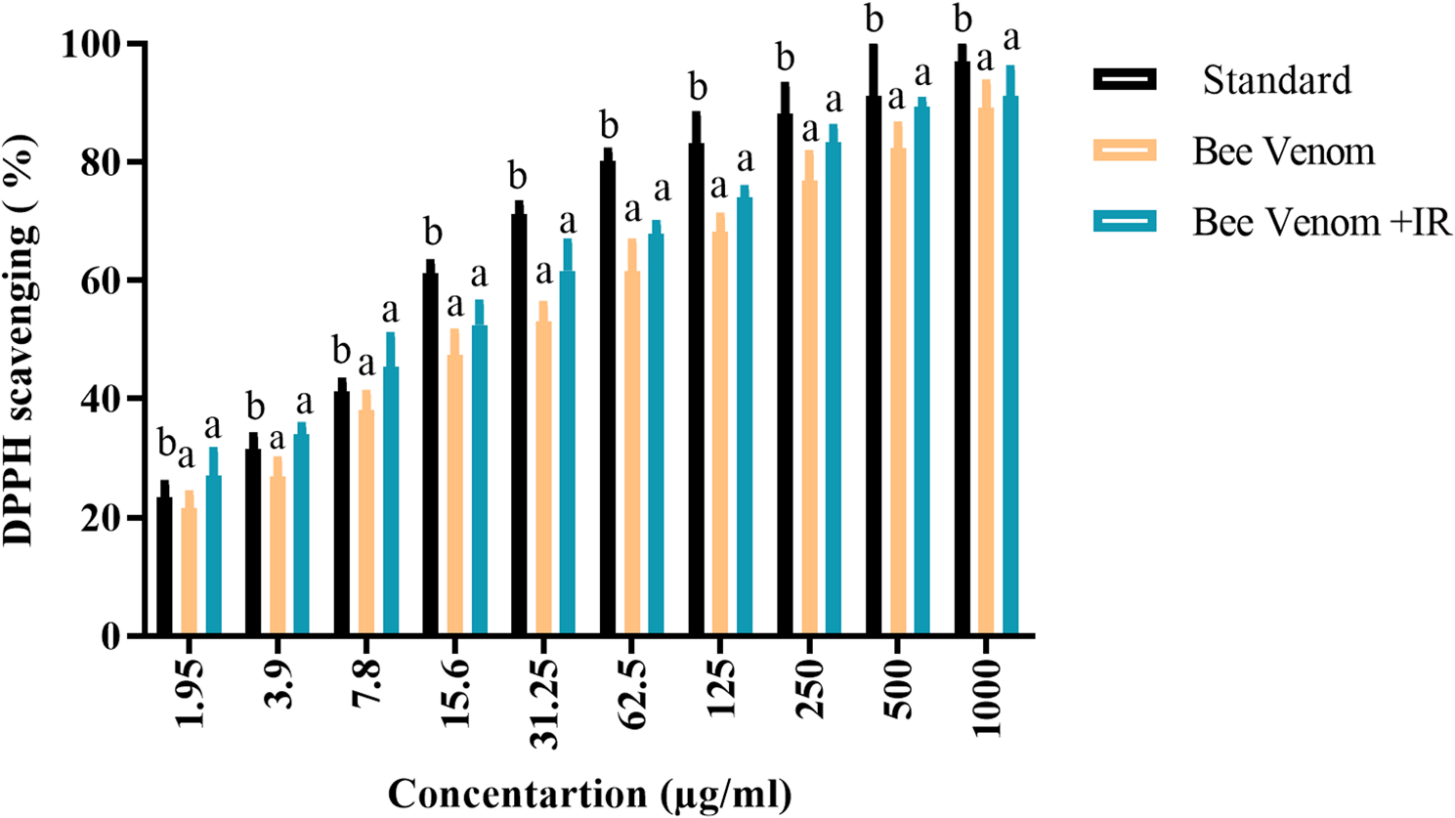
Antioxidant capacity of bee venom (BV) and infrared-irradiated BV compared with ascorbic acid (standard). Data are reported as mean ± SD; [analysis done by One-way Analysis of Variance (ANOVA), followed by post-hoc test, different letters above bars indicate significant differences at *p* ≤ 0.05

The native BV extract demonstrated appreciable radical-scavenging activity, with a DPPH assay IC₅₀ of 27.65 ± 0.8 µg/mL (95% CI 26.1–29.2; R^2^ = 0.98, n = 3). IR irradiation significantly enhanced the antioxidant capacity, reducing the IC₅₀ to 16.47 ± 1.1 µg/mL (95% CI 14.3–18.6; R^2^ = 0.97, n = 3). For context, the standard antioxidant ascorbic acid exhibited a substantially lower IC₅₀ of 2.95 ± 0.2 µg/mL (95% CI 2.6–3.3; R^2^ = 0.99, n = 3) (Fig. 6).

The antioxidant effects of BV are likely attributable to the synergistic actions of its volatile constituents, peptides, and other bioactive molecules. The impact of IR on antioxidant properties is variable and depends on the chemical composition of the sample and the irradiation parameters; the literature documents heterogeneity, with reports of decreased activity, no significant change, or context-specific enhancement in antioxidant metrics (Campos et al. 2021; Isidorov et al. 2023). Notably, the present study demonstrates that a brief, energy-efficient IR treatment can lower the DPPH IC_50_ of BV by approximately ~ 40%, indicating a substantial enhancement in radical-scavenging efficiency without altering the extract's overall chemical profile.

### Anti-inflammatory activity (COX-1/COX-2)

**Fig. 7:**
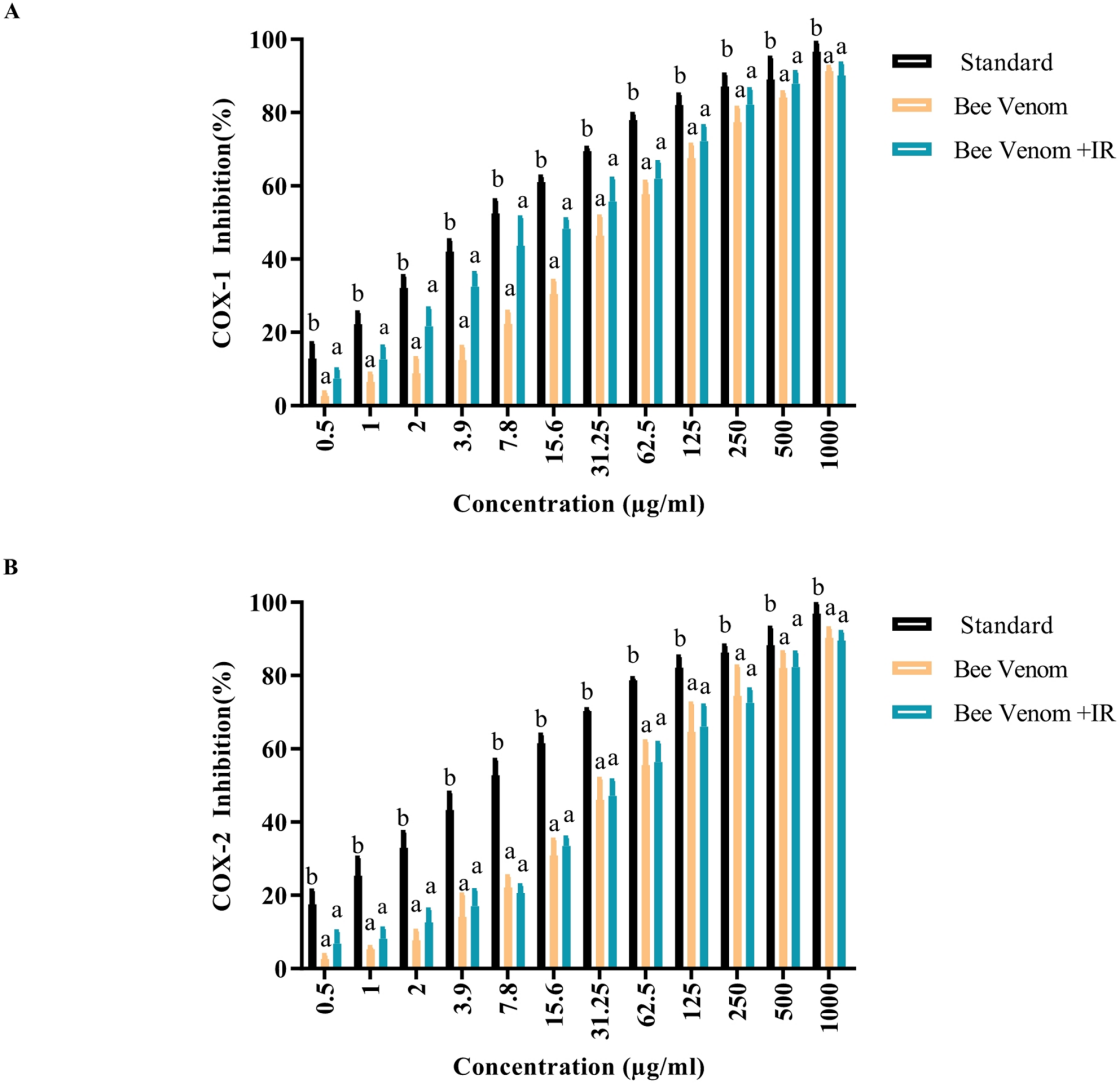
Anti-inflammatory activity of bee venom (BV) and infrared-irradiated BV relative to celecoxib (standard). **A** COX-1 inhibition. **B** COX-2 inhibition. Data are reported as mean ± SD; [analysis done by One-way Analysis of Variance (ANOVA), followed by post-hoc test; different letters above bars indicate significant differences at *p* ≤ 0.05

BV inhibited COX-1 and COX-2 with IC₅₀ values of 41.74 ± 0.2 and 50.99 ± 0.9 µg/mL, respectively. IR irradiation reduced the IC₅₀ values to 24.70 ± 0.2 µg/mL for COX-1 and 48.84 ± 0.2 µg/mL for COX-2, indicating a measurable but modest improvement in inhibitory potency, particularly toward COX-1. For comparison, a standard inhibitor exhibited substantially lower IC₅₀ values of 7.40 ± 0.5 and 7.71 ± 0.3 µg/mL for COX-1 and COX-2, respectively (Figs. 7a and b), highlighting that BV, with or without IR treatment, remains considerably less potent than established pharmacological inhibitors. These findings suggest that IR treatment fine-tunes the inhibitory profile of BV rather than dramatically improving its overall potency.

COX-1 is constitutive and supports homeostatic functions, such as gastric mucosal protection and renal blood flow, whereas cyclooxygenase-2 (COX-2) is primarily induced during inflammation and tissue injury (Bishayee et al. 2024). The aqueous fraction of BV has been reported to exert dose-dependent COX-2 suppression, often with limited effects on COX-1 (Saad 2025). While some natural extracts inhibit both isoforms, IR irradiation has been shown to modulate COX-2 activity; for instance, radioprotective strategies in oncology have explored combining IR modalities with COX-2 inhibitors (Chahal et al. 2023; Ketha et al. 2020).

### Anticancer activity

**Fig. 8:**
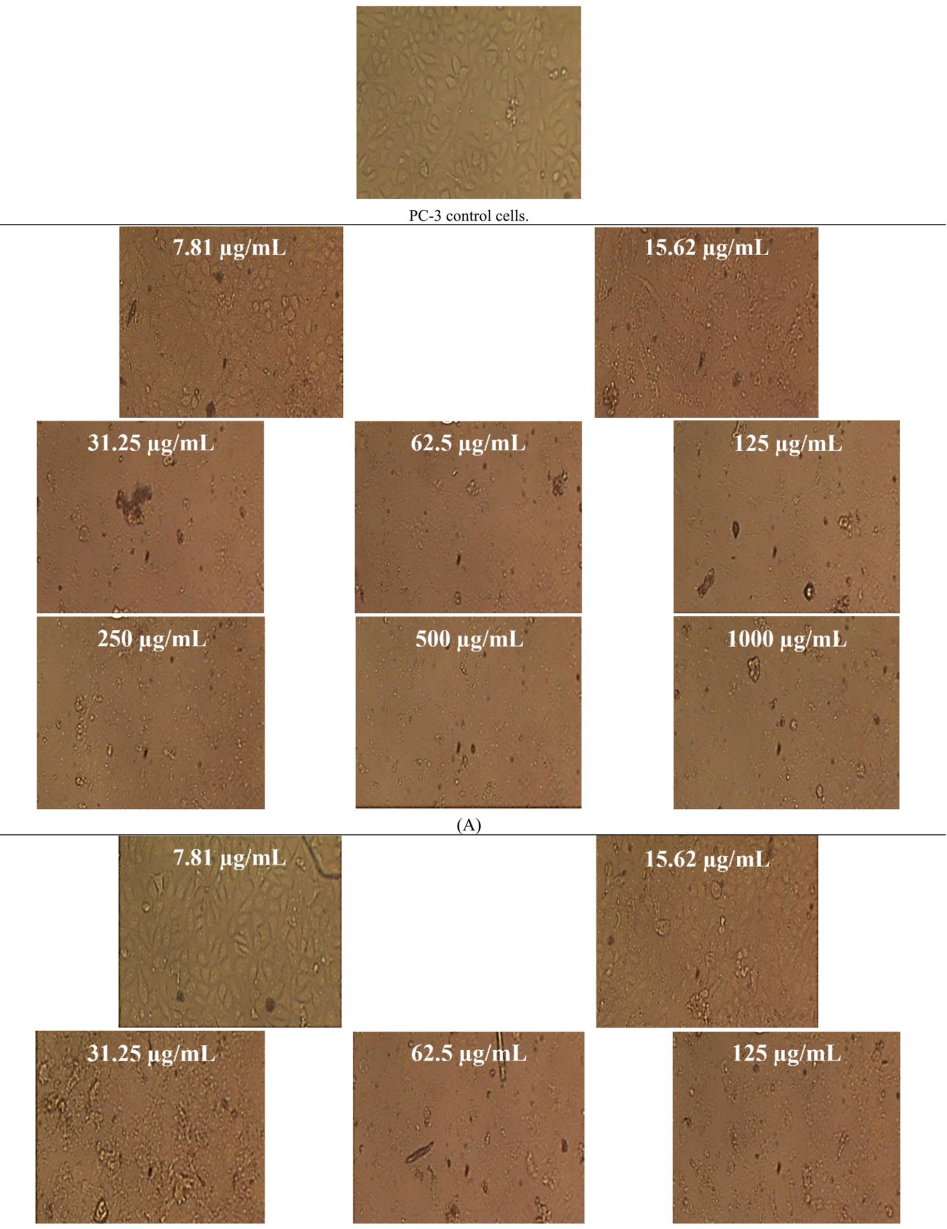

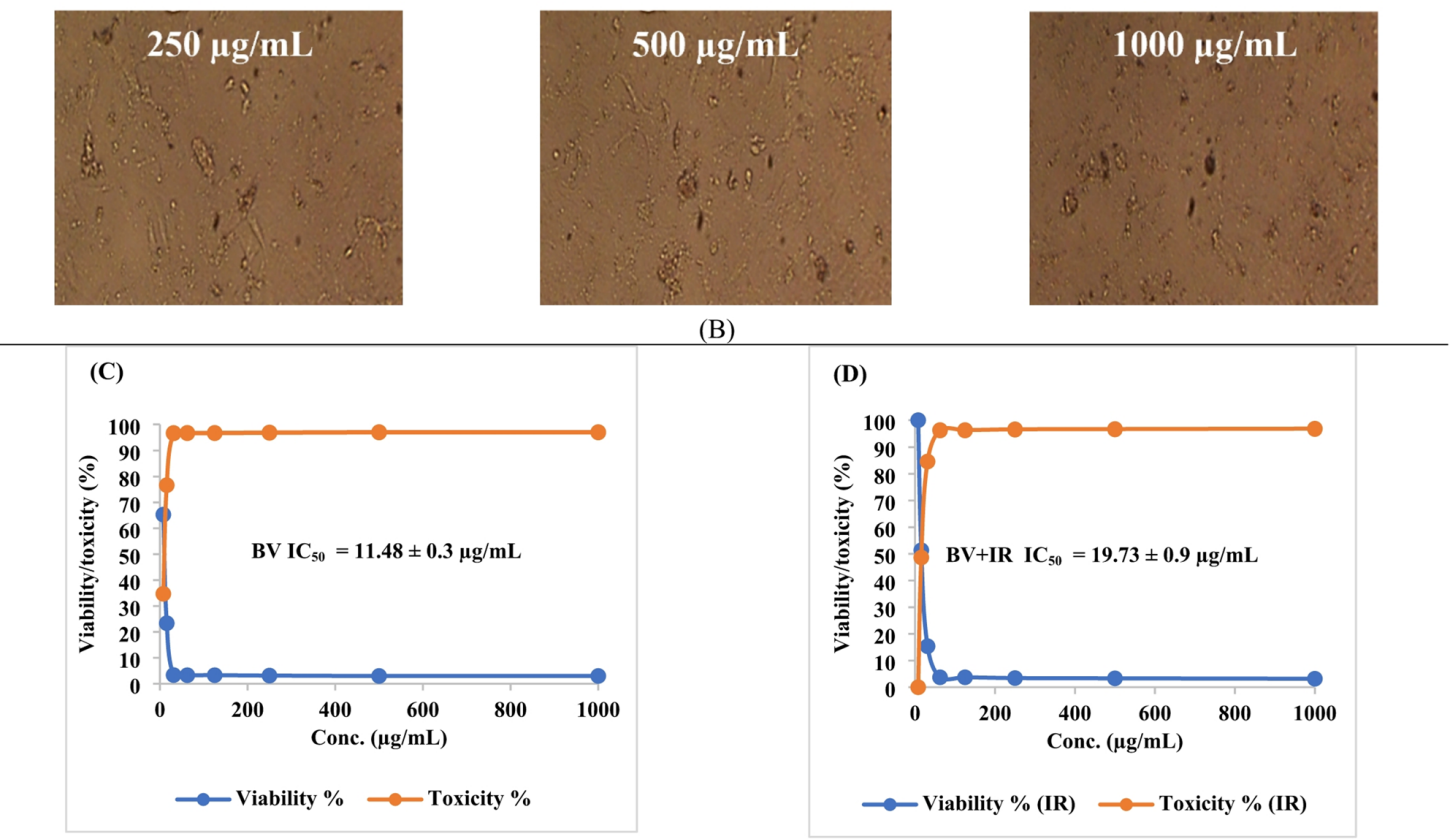
Microscopic assessment of anticancer activity against PC-3 cells for **A** non-irradiated bee venom (BV) and **B** infrared-irradiated BV across 31.25–1,000 µg/mL (magnification, 40 ×). **C**, **D** Quantitative analyses of cell viability (%) and cytotoxicity (%) for both BV forms over the tested concentrations. Data are reported as mean ± SD

**Fig. 9:**
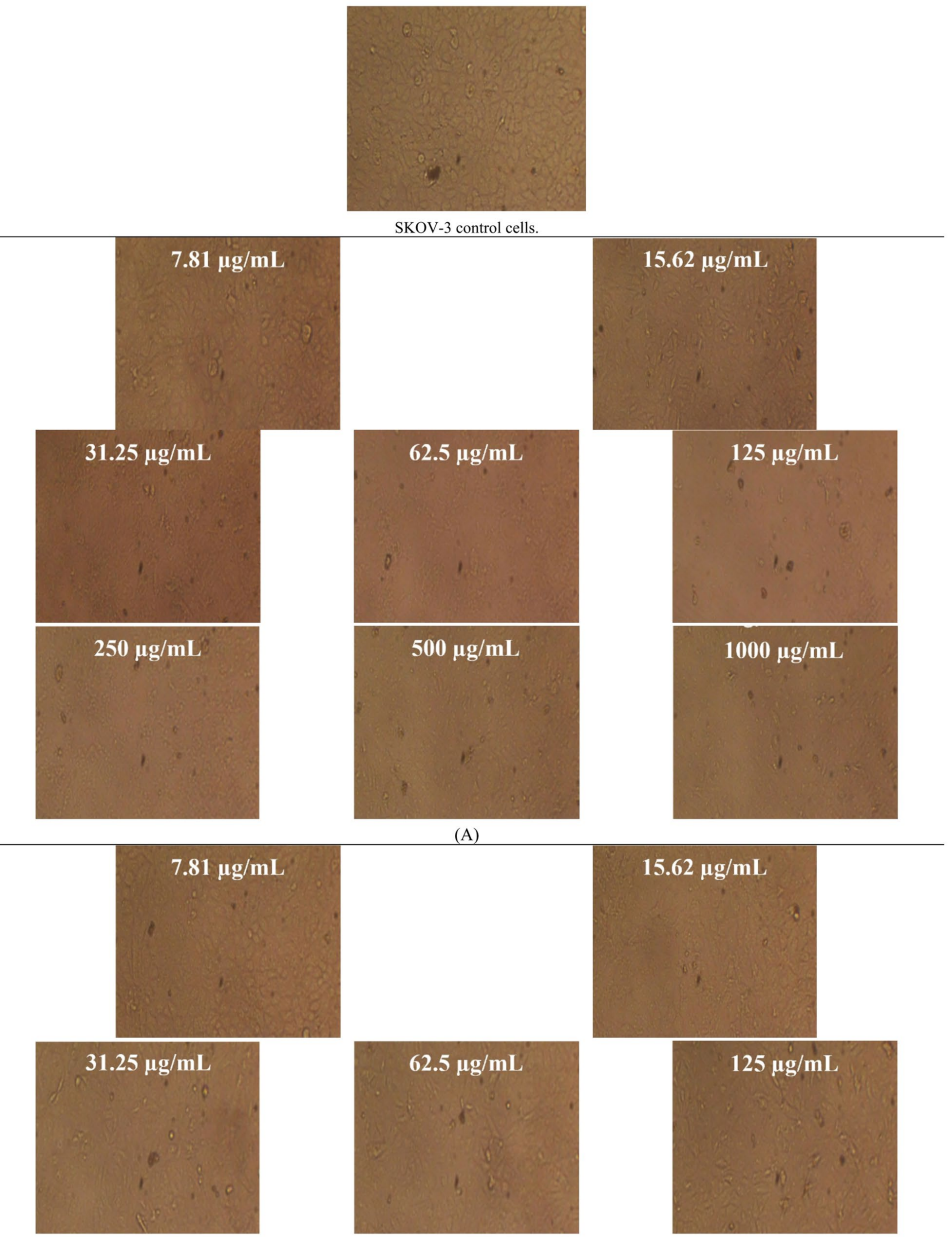

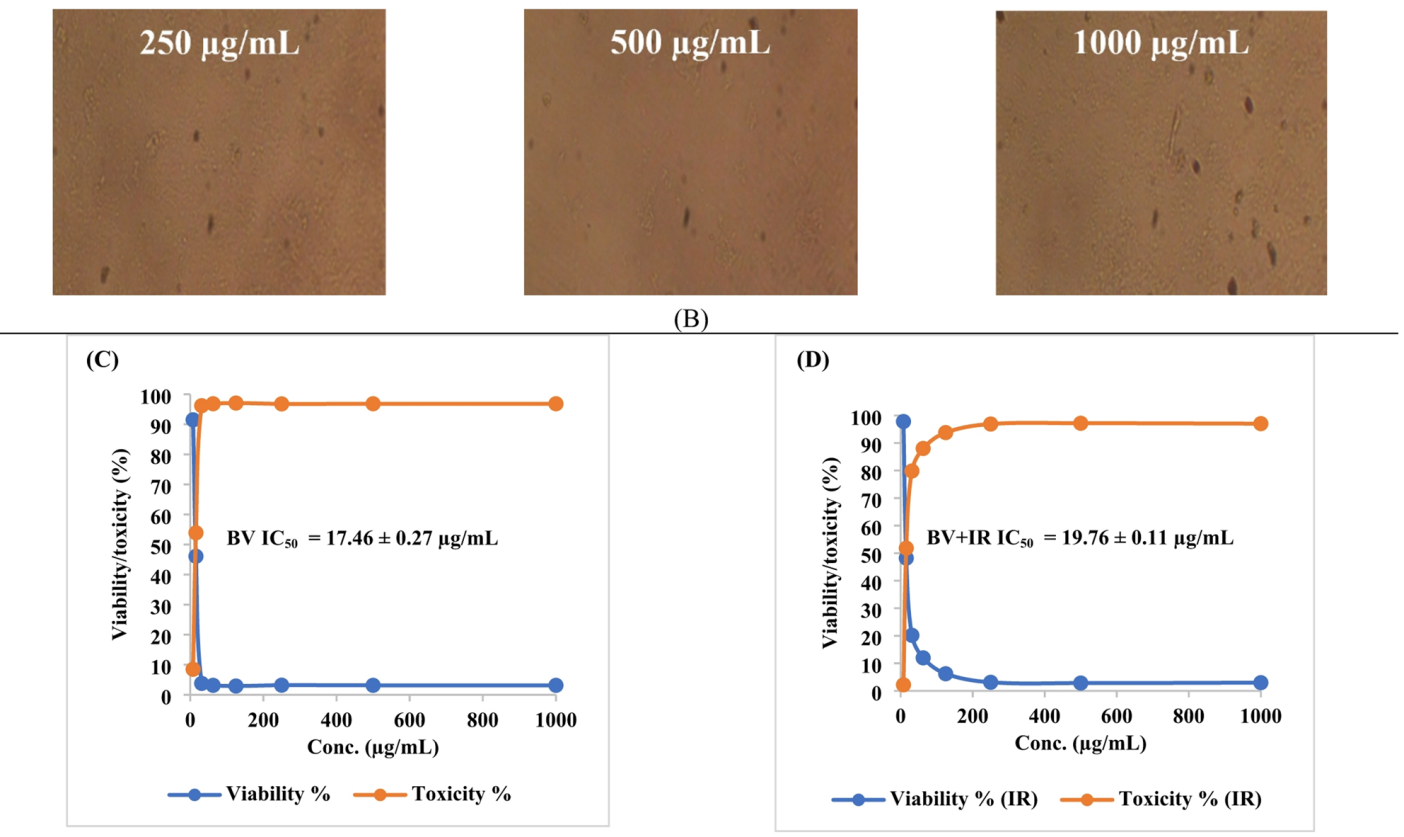
Microscopic assessment of antitumor activity against SKOV-3 cells for **A** non-irradiated bee venom (BV) and **B** infrared-irradiated BV across 31.25–1,000 µg/mL (magnification, 40 ×). **C**, **D** Quantitative analyses of cell viability (%) and cytotoxicity (%) for both BV forms over the tested concentrations. Data are reported as mean ± SD

In this study, native BV demonstrated cytotoxicity against PC-3 (prostate cancer) and SKOV-3 (ovarian cancer) cell lines, with IC₅₀ values of 11.48 ± 0.3 µg/mL (95% CI 10.9–12.1; Hill slope =  − 1.21) and 17.46 ± 0.27 µg/mL (95% CI 16.9–18.0; Hill slope =  − 1.08), respectively. Following IR irradiation, the cytotoxic activity of BV was reduced, yielding IC₅₀ values of 19.73 ± 0.9 µg/mL (95% CI 18.1–21.4; Hill slope =  − 0.94) for PC-3 cells and 19.76 ± 0.11 µg/mL (95% CI 19.5–20.0; Hill slope =  − 0.97) for SKOV-3 cells, indicating a quantifiable decrease in potency rather than an enhancement (Figs. 8, 9 and Tables 4, 5). All IC₅₀ values were derived from fitted nonlinear dose–response curves using, and corresponding Hill slopes and confidence intervals are reported to support the robustness of the cytotoxicity analysis. These results indicate a clear decrease in cytotoxic potency rather than an enhancement of anticancer activity.

**Table 4:** Anticancer activity of bee venom (BV) before and after infrared (IR) irradiation (BV + IR) against PC-3 cells. Data are reported as mean ± SD

ID	Conc. (µg/mL)	O.D			Mean O.D	±SE	Viability (%)	Toxicity (%)	IC_50_ ± SD (µg/mL)
PC-3	--------	0.68	0.676	0.687	0.681	0.003215	100	0	
BV	7.81	0.452	0.444	0.438	0.444667	0.004055	65.29613314	34.70386686	11.48 ± 0.3
	15.62	0.165	0.153	0.159	0.159	0.003464	23.34801762	76.65198238	
	31.25	0.023	0.022	0.023	0.022667	0.000333	3.328438571	96.67156143	
	62.5	0.022	0.024	0.021	0.022333	0.000882	3.279490945	96.72050906	
	125	0.022	0.022	0.023	0.022333	0.000333	3.279490945	96.72050906	
	250	0.023	0.019	0.022	0.021333	0.001202	3.132648067	96.86735193	
	500	0.021	0.02	0.02	0.020333	0.000333	2.985805188	97.01419481	
	1000	0.02	0.019	0.022	0.020333	0.000882	2.985805188	97.01419481	
BV + IR	7.81	0.682	0.68	0.681	0.681	0.000577	100	0	19.73 ± 0.9
	15.62	0.35	0.348	0.351	0.349667	0.000882	51.34605972	48.65394028	
	31.25	0.096	0.108	0.11	0.104667	0.004372	15.36955458	84.63044542	
	62.5	0.025	0.027	0.024	0.025333	0.000882	3.720019579	96.27998042	
	125	0.026	0.024	0.026	0.025333	0.000667	3.720019579	96.27998042	
	250	0.026	0.023	0.021	0.023333	0.001453	3.426333823	96.57366618	
	500	0.023	0.023	0.022	0.022667	0.000333	3.328438571	96.67156143	
	1000	0.02	0.021	0.024	0.021667	0.001202	3.181595693	96.81840431	

**Table 5:** Antineoplastic activity of bee venom (BV) before and after infrared (IR) irradiation (BV + IR) against SKOV-3 cells. Data are reported as mean ± SD

ID	Conc. (µg/mL)	O.D			Mean O.D	±SE	Viability (%)	Toxicity (%)	IC_50_ ± SD (µg/mL)
SKOV-3	--------	0.729	0.733	0.725	0.729	0.002309	100	0	
BV	7.81	0.684	0.651	0.666	0.667	0.009539	91.4951989	8.504801097	17.46 ± 0.27
	15.62	0.342	0.33	0.336	0.336	0.003464	46.09053498	53.90946502	
	31.25	0.029	0.03	0.024	0.027667	0.001856	3.795153178	96.20484682	
	62.5	0.024	0.022	0.023	0.023	0.000577	3.155006859	96.84499314	
	125	0.021	0.023	0.02	0.021333	0.000882	2.926383173	97.07361683	
	250	0.023	0.025	0.022	0.023333	0.000882	3.200731596	96.7992684	
	500	0.022	0.022	0.025	0.023	0.001	3.155006859	96.84499314	
	1000	0.024	0.022	0.023	0.023	0.000577	3.155006859	96.84499314	
BV + IR	7.81	0.708	0.718	0.713	0.713	0.002887	97.80521262	2.19478738	19.76 ± 0.11
	15.62	0.347	0.352	0.355	0.351333	0.002333	48.19387289	51.80612711	
	31.25	0.144	0.156	0.141	0.147	0.004583	20.16460905	79.83539095	
	62.5	0.084	0.09	0.088	0.087333	0.001764	11.97988112	88.02011888	
	125	0.036	0.053	0.047	0.045333	0.004978	6.218564243	93.78143576	
	250	0.023	0.022	0.023	0.022667	0.000333	3.109282122	96.89071788	
	500	0.02	0.019	0.023	0.020667	0.001202	2.834933699	97.1650663	
	1000	0.022	0.022	0.021	0.021667	0.000333	2.97210791	97.02789209	

The persistent global burden of cancer underscores the critical need for effective and safer therapeutic strategies (Hanahan 2022; Tran et al. 2022). BV has been investigated in apitherapy for its diverse pharmacological properties, including analgesic, anti-inflammatory, antimutagenic, radioprotective, and anticancer effects (Erkoc et al. 2022). The processing of natural extracts with IR has previously been reported to enhance bioactivities such as antioxidant capacity and, in certain contexts, anticancer efficacy (Azad et al. 2018). However, such effects are not universal and appear to be highly context- and target-dependent. Indeed, natural products remain a vital source of leads for novel anticancer agents and mechanisms of action (Chunarkar-Patil et al. 2024). In the present study, IR irradiation did not improve BV-mediated cytotoxicity and instead resulted in attenuated activity against both cancer cell lines. While this treatment reduced the disparity in IC₅₀ values between PC-3 and SKOV-3 cells, this convergence reflects a loss of potency rather than a therapeutic gain. Accordingly, IR irradiation should be interpreted as altering, not enhancing, the anticancer profile of BV, and the biological implications of this attenuation warrant further mechanistic investigation before therapeutic relevance can be inferred. The observed bioactivity enhancement is driven by two synergistic mechanisms: chemical enrichment and structural bio-activation. The enrichment of the benzopyran-4-one derivative accounts for the increased biological capacity. Meanwhile, IR irradiation likely induces conformational changes in the peptide fraction, significantly altering biological activities. These peptide modifications are not detected by GC–MS but enhance membrane disruption. Ultimately, the combined effect of enriched volatiles and physically activated peptides may explain the broad functional improvements.

## Conclusions

This study establishes brief IR pretreatment as a promising preliminary method for augmenting the in vitro functional properties of the low-molecular-weight fraction of bee venom. This treatment distinctly enriched the concentration of key bioactive constituents, particularly a benzopyran-4-one derivative. Concomitantly, IR irradiation resulted in consistent, albeit modest, enhancements in multiple functional properties: antimicrobial and antibiofilm potency, radical-scavenging capacity, cyclooxygenase (COX-1 and COX-2) inhibition, and cytotoxic activity against PC-3 and SKOV-3 cancer cell lines. The antihemolytic activity was also modulated in a pathogen- and concentration-dependent manner. These collective findings underscore the utility of IR irradiation as a straightforward, energy-efficient, and solvent-free post-processing strategy to refine the chemical composition of BV and selectively enhance its bio-functional properties. Future work should focus on the systematic optimization of irradiation parameters, stability testing, and complementary peptide-focused analytical techniques to maximize potential therapeutic efficacy and safety to maximize therapeutic efficacy and safety. These collective findings underscore the utility of IR irradiation as a straightforward, energy-efficient, and solvent-free post-processing strategy to refine the volatile chemical composition of BV and selectively enhance its bio-functional properties within the scope of this study. However, it is important to note that these chemical observations are limited to the volatile fraction and do not account for potential changes in the peptide or protein profile. Additionally, while cytotoxicity and antimicrobial activity were observed, further validation through mechanistic assays (e.g., Annexin V/PI) and in vivo studies is required to confirm therapeutic relevance. The limitations of this study include: (1) bee venom is composed of peptides and proteins that are not amenable to GC–MS analysis. Accordingly, the chemical changes detected following IR irradiation had been interpreted as alterations within the low-molecular-weight fraction only. No peptide- or protein-specific analytical methods (e.g., LC–MS/MS or proteomic profiling) were performed in this study; therefore, no conclusions can be drawn regarding quantitative or qualitative changes in major BV peptides. Future studies incorporating complementary peptide-focused analytical techniques will be required to comprehensively assess the impact of IR irradiation on the dominant bioactive constituents of BV. (2) The agar well diffusion assay is influenced by physicochemical properties such as sample viscosity, molecular size, and diffusion capacity within the agar matrix. In this study, both native and IR-irradiated BV samples were tested as crude extracts using the same solvent, concentration, well diameter, and incubation conditions to ensure direct comparability. Nevertheless, differences in diffusion behavior following IR treatment cannot be fully excluded and may contribute to variations in inhibition zone diameters. Accordingly, zone-of-inhibition results were interpreted qualitatively and in conjunction with broth-based MIC determinations rather than as standalone quantitative measures of antimicrobial potency. (3) Specific viscosity, thermodynamic or photochemical explanation measurements were not performed and remain a target for future validation.

## Data Availability

The datasets generated during the current investigation are available from the corresponding author on reasonable request.

## Abbreviations

BV: Bee venom
HBV: Honeybee venom
GC–MS: Gas chromatography-mass spectrometry
IR: Infrared
MIC: Minimum inhibitory concentration
MBC: Minimum bactericidal concentration
MHA: Mueller–Hinton agar
MEA: Malt extract agar
RBCs: Red blood cells
COX-1/COX-2: Cyclooxygenase-1/-2
PC-3: Human prostate cancer cells
SKOV-3: Human ovarian cancer cells
